# Gaining Micropattern Fidelity in an NOA81 Microsieve Laser Ablation Process

**DOI:** 10.3390/mi12010021

**Published:** 2020-12-27

**Authors:** Rahman Sabahi-Kaviani, Regina Luttge

**Affiliations:** Neuro-Nanoscale Engineering, Mechanical Engineering Department and Institute of Complex Molecular Systems, Eindhoven University of Technology (TU/e), 5600MB Eindhoven, The Netherlands; r.sabahi.kaviani@tue.nl

**Keywords:** microsieve, replica molding, NOA81, excimer laser micromachining

## Abstract

We studied the micropattern fidelity of a Norland Optical Adhesive 81 (NOA81) microsieve made by soft-lithography and laser micromachining. Ablation opens replicated cavities, resulting in three-dimensional (3D) micropores. We previously demonstrated that microsieves can capture cells by passive pumping. Flow, capture yield, and cell survival depend on the control of the micropore geometry and must yield high reproducibility within the device and from device to device. We investigated the NOA81 film thickness, the laser pulse repetition rate, the number of pulses, and the beam focusing distance. For NOA81 films spin-coated between 600 and 1200 rpm, the pulse number controls the breaching of films to form the pore’s aperture and dominates the process. Pulse repetition rates between 50 and 200 Hz had no observable influence. We also explored laser focal plane to substrate distance to find the most effective ablation conditions. Scanning electron micrographs (SEM) of focused ion beam (FIB)-cut cross sections of the NOA81 micropores and inverted micropore copies in polydimethylsiloxane (PDMS) show a smooth surface topology with minimal debris. Our studies reveal that the combined process allows for a 3D micropore quality from device to device with a large enough process window for biological studies.

## 1. Introduction

Laser micromachining has gained popularity as a fabrication method for microsystems that need specific materials to be processed [1,2,3,4,5]. Such a requirement can also be found in the quest for advanced 3D tissue models [6,7,8,9,10,11]. Several studies have demonstrated the importance of mechanical properties such as stiffness and curvature in the design of in vitro cell cultures [12,13,14]. The design of proper 3D microenvironments enhances cell adherence and maintains the cells’ natural spherical 3D morphology [12,15,16]. Among other authors in the literature [17,18], we have also shown that there is a difference in neuronal differentiation and organization as well as total neurite length between 2D and 3D cell cultures [19,20,21]. In this work, we focus on the optimization of a microfabrication process for a microsieve structure, i.e., arrayed 3D micropores. Originally, these microsieves were made with a high pattern fidelity by silicon micromachining, as we demonstrated to pair single cells to electrodes [22]. This technique was developed by Schurink et al. with a high potency for cell capture yield and survival [23]. Additive manufacturing using photo-curable polymers is an alternative for photo-lithography processes and can directly yield a final product structure, which allows for the implementation of complex 3D-multilayer patterns in a cost-accessible manner [24,25,26,27]. Such capabilities have been explored for breast cancer cell culture [28]. Furthermore, fabrication of 3D multilayers patterns for neuronal culture using photo curable materials has rapidly received attention [29,30]. Therefore, lately, we also demonstrated a microsieve structure that was similar to that developed by Schurink et al. but was made from a photo-curable optical adhesive (NOA81), and it showed an even higher cell survival than silicon [31]. Microsieves made from polymer materials, specifically using NOA81, known as an optical adhesive, offer new capabilities in this field of application. NOA81 micromachining took place via drop-casting in double replica molding and subsequent excimer laser ablation, resulting in funnel-like 3D micropores. Generally, it is known that the material’s properties and specific geometries of the microenvironment are important factors in cell differentiation [32]. In this paper, we introduce the control of parameters of the replica molding and laser ablation processes for 3D micropores in more depth.

Consequently, we investigated polydimethylsiloxane (PDMS)-to-NOA81 replica molding via spin-coating and ultraviolet (UV) exposure to create NOA81 microcavities in the polymeric foils of a range of defined thicknesses, and thus prepared patterned substrates for ablation tests to control the specific 3D geometry of the pores. For all ablation tests, a krypton fluoride (KrF) nanosecond excimer laser setup was used in order to investigate how the 3D micropore’s geometry depends on the pulse repetition frequency, the number of pulses, and the laser beam focus distance adjustment relative to the patterned substrate surface. The new results found in this study resemble our previous laser ablation results in NOA81 but improve upon the reproducibility of the microfabrication process. This method of micromachining allows for a rapid prototyping approach to further explore design features for microsieve-assisted cell capturing.

## 2. Materials and Methods

### 2.1. NOA81 Replica Molding from Silicon Microsieve Electrode Array

**Figure 1:**
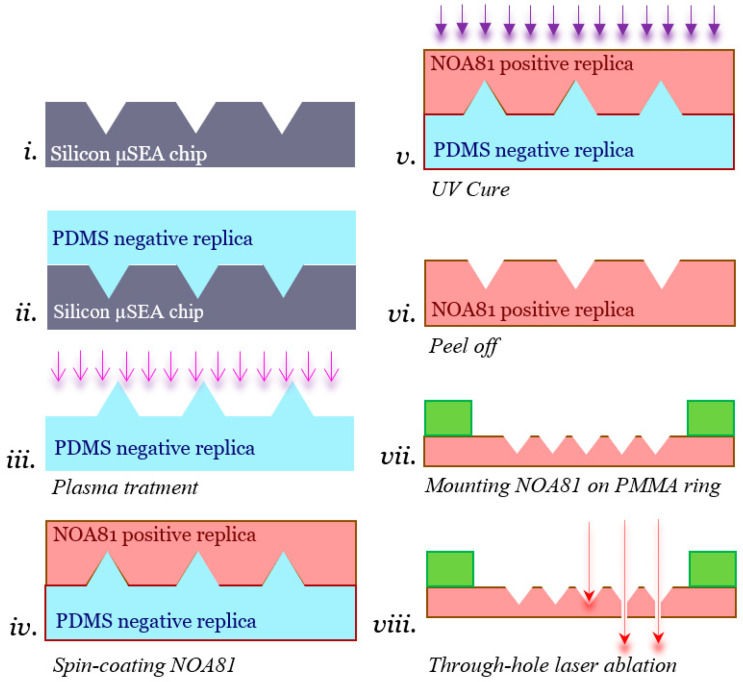
Replica molding and laser ablation process workflow for NOA81 microsieve. From a clean silicon electrode microsieve array (μSEA), (**i**) a negative replica is created by casting polydimethylsiloxane (PDMS) (10:1 *w*/*w* ratio) followed by curing at 95 °C and peeling off (**ii**). The PDMS mold is then oxygen-plasma-treated with 10 W for 30 s to become hydrophilic and allowing for a wetting of NOA81 during spinning (**iii**). An excess of NOA81 is poured onto the mold and spin-coated at 500 rpm for 30 s followed by whatever speed results in the final thickness for 60 s (**iv**). The NOA81 is then cured under ultraviolet (UV) light with an energy amount of 2100 mJ/cm^2^ at an intensity of 15 mW/cm^2^ (**v**). NOA81 is then peeled off quickly (**vi**) and is mounted on a polymethyl methacrylate (PMMA) ring (**vii**). The assembly is then submitted to an excimer laser station to produce the through-holes in the pyramid structures by ablation (**viii**).

**Figure 2:**
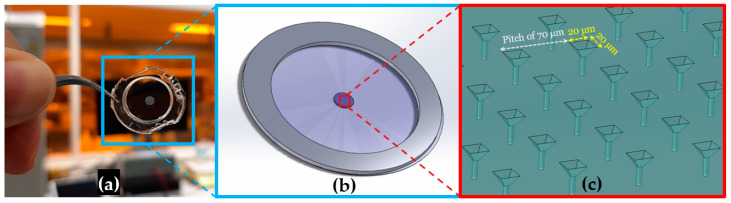
(**a**) A fabricated NOA81 foil mounted on a PMMA ring for ease of handling. All the 900 pyramid-shaped microcavities are in the central circle with a diameter of 2.4 mm, of which a selection is subsequently ablated to form the arrayed 3D micropores. (**b**) A 3D SOLIDWORKS sketch of the resulting microsieve chip. (**c**) Zoom-in of the anticipated 3D micropore design at scale.

NOA81 (Norland Optical Adhesive 81) microsieves were fabricated by a double replica molding procedure fully described by Moonen et al. [31]. In brief, the microsieve pattern of an original micro-Silicon Electrode Array (µSEA) fabricated previously by Schurink during his PhD work at University of Twente [33] was transferred into polydimethylsiloxane (PDMS) using standard soft lithography and NOA81 replica molding. In order to better appreciate improvements yielded in this paper, the fabrication process is depicted in Figure 1 for completeness. First, the μSEA, here used as a master mold, was blow-cleaned using an N_2_ gun (Figure 1i). Afterwards, a PDMS base and cross-linking agents (SYLGARD 184, Dow Corning, Midland, MI, USA), i.e., the two liquid components of PDMS, were mixed at a ratio of 10:1, and the mixture was placed inside a vacuum desiccator for 20 min to degas. After removal of the air bubbles, the mixture was poured on the µSEA with a thickness of around 500 µm, and the PDMS was cured by placing the μSEA on a hot plate at 95 °C for 10 min (Figure 1ii). The PDMS was peeled off from the master mold and cut into a square of 2 × 2 cm^2^, acting as an inverted mold for the subsequent NOA81 replica molding step. Here, we investigated spin-coating instead of drop-casting to yield a well-defined NOA81 film thickness for ensuing detailed laser ablation studies. Since the PDMS mold needs to be hydrophilic to allow a wetting of NOA81 during spin-coating, a plasma oxidation step at 10 W for 30 s was performed using a plasma asher (EMITECH K1050X, Quorum, Laughton, UK) (Figure 1iii). NOA81 liquid was poured in excess on the surface of the PDMS mold shortly after plasma activation covering the full area of the mold. Next, spin-coating was performed in three consecutive steps: first at 500 rpm for 30 s with an acceleration of 200 rpm/s, then at a speed depending on the desired final thickness for 60 s (Figure 1iv) and with an acceleration of 300 rpm/s, and finally with a deceleration of 300 rpm/s until it stopped. The mold with the NOA81 film was placed inside a UV-LED exposure system (IDONUS, UV-EXP 150R, Neuchatel, Switzerland) with an intensity set to 15 mW/cm^2^. Pre-curing of the NOA81 films was achieved when they received a UV dosage yielding 2100 mJ/cm^2^ (Figure 1v). A higher amount of UV dosage or higher intensity hampers the separation between PDMS and NOA81, and a lower dosage will not sufficiently pre-cure it for peeling. Although UV dosage may also depend on the thickness of photo-cured films, we have not observed this effect in the range of thicknesses that we have produced with NOA81. After photo-curing, the NOA81 film was therefore peeled off quickly (Figure 1vi). A delay in this step can lead to a strong bond and make peeling off impossible. During the peeling-off step, the thin NOA81 can bend or roll over, so the process should be practiced with caution. After peeling the film off the PDMS mold, the resulting thin NOA81 foil contained the microcavities and was placed on a flat substrate, such as on a paper tissue atop of a glass slide, with as little wrinkles as possible, and put back inside the UV exposure system once more with the above-mentioned settings to be completely cured. To assess how the thickness of patterned NOA81 foils depends on spin-coating speeds, the thickness of the cured polymer foil was measured using a profilometer (HEIDENHAIN, Traunreut, Germany). In this step, thickness measurements were performed on a flat region close to the cavities. For an easier handling of the NOA81 foil during laser micromachining and cell culture, the foil was mounted on a ring made of polymethyl methacrylate (PMMA) with a thickness of either 0.5 mm or 1 mm and an inner and an outer diameter of 13 mm and 18 mm, respectively (Figure 1vii). The two pieces were attached by transferring a small amount of NOA81 onto the PMMA ring using a sharp tip and placing the foil on it. The two pieces were then assembled firmly after a UV light curing step with the settings previously mentioned. It is important to keep in mind which side of the polymer chip has the microcavity structures when mounting the foil on the ring, which serves as a simple holder. The last step in the process was the laser ablation, to yield the microsieve with its arrayed 3D micropores. This step is discussed in Section 2.2, and all results per step are presented in Section 3. An example of a fabricated microsieve mounted on the holder is shown in Figure 2a. A 3D SOLIDWORKS screenshot depicts the assembly with 900 square pyramidal-shaped microcavities laying within a circular area with a 2.4 mm diameter in the center in Figure 2b, and a zoom-in thereof shows the pyramidal shape and spacing of the micropores at scale in Figure 2c. In our design, the pyramidal structure extends at its tip into a uniform diameter through-hole. Its diameter is indicative of the exit hole size, which corresponds to a 3D micropore geometry that is expected to capture an individual cell.

### 2.2. Laser Ablation of the NOA81 Microsieve

The NOA81 foils containing the microcavities, which were mounted on a PMMA holder, were placed inside the Optec MicroMaster KrF-Laser set-up (Optec S.A., Frameries, Belgium) that produces ultraviolet light with a wavelength of 248 nm and a pulse duration of 5–6 ns for laser ablation treatment (Figure 1viii) [34]. In this step, the laser beam is positioned at the location of the tip of a pyramid, i.e., in the center of the microcavity, and the laser generates a through-hole. Afterwards, this process is repeated until the required number of through-holes forming the microsieve is achieved. The tool is equipped with a servo motor control that can produce movements of 1 µm in all three coordinates, and the so-called LightDeck system allows us to define the ablation position relative to the surface of the foil within the center of the prepatterned microcavities. The pulse energy is kept constant over time by the tool’s automatic laser voltage adjustment up to a maximum limit, and the gas must be exchanged for consistency in the machining process thereafter. Although in this class of lasers the thermal damage is minimal per pulse, ablation in the thermal and the plasma regime can take place. The size of the generated laser beam after passing the de-magnifying objective can be reduced to a diameter of at least 10 µm. Given the size of neuronal cells, which is also around a diameter of 10 µm, the resultant opening using this laser beam size depends also on the ablation depths and could be wider than required for efficient capturing cells by passive capillary flow [31]. To explore which parameters of the laser ablation process affect the geometry and quality of the resulting feature dependent on the NOA81 film thickness, several different settings have been tested. These include the pulse repetition frequency, the number of laser pulses, and the vertical distance of the surface of NOA81 foil to the height where the beam is focused by visual inspection. The recommended working range of frequency of the laser is up to 150 Hz; however, to investigate the effect of this parameter, we performed experiments with 50, 100, 150, and 200 Hz. Furthermore, the number of laser pulses on each position can be increased to even tens of thousands of pulses; however, only increasing this parameter will not increase the ablated depth without refocusing the laser beam in z-direction. Our results and discussion regarding the ablation parameters for gaining micropattern fidelity in the fabrication process for microsieves are further refined in Section 3.

## 3. Results and Discussion

### 3.1. NOA81 Device Replication

**Figure 3:**
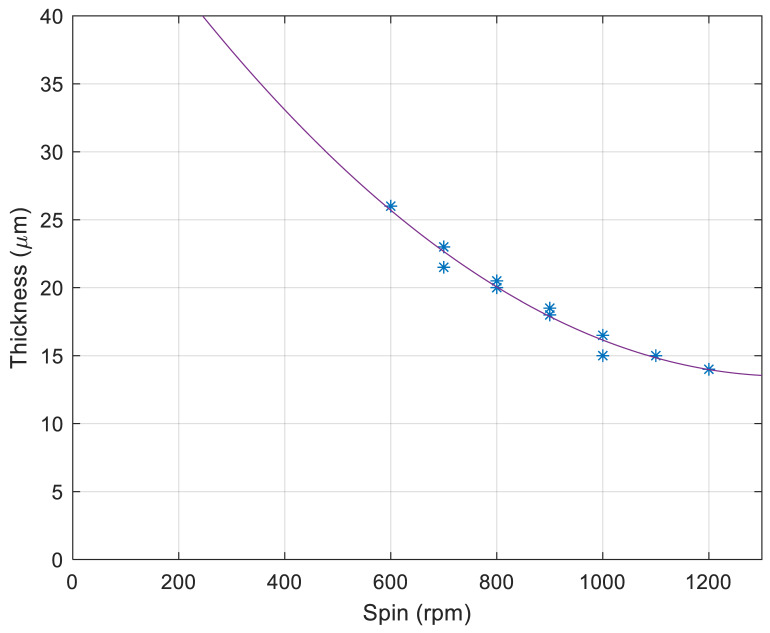
NOA81 foil thickness against spin-coating speeds. The curve is a quadratic fit to the data. The function equation of the curve is $y=2.2\times 10^{-5}x^{2}-0.059x+53$ and the R-squared value for the fitting curve is 0.978.

**Figure 4:**
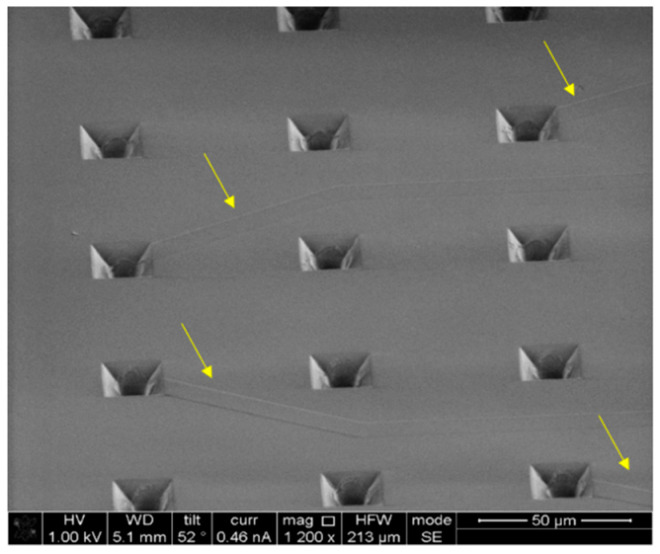
Fabricated NOA81 microsieve using spin-coating shows a successful replica with the nanometer high features of the original μSEA (yellow arrows).

The material used in this article for the fabrication of microsieves is NOA81. It is a single component adhesive liquid that will cure within a few seconds to a few minutes to a tough and hard polymer when exposed to ultraviolet (UV) light [35]. This polymer was previously proposed as an alternative for PDMS in microfluidic chip applications [36] and has been used for low-cost microfluidic and microchannel fabrication [37]. In this work, the double replica molding procedure with PDMS as the negative copy of the original μSEA and NOA81 as the final device material was improved to achieve a certain thickness of the polymer microsieve structure. In a previous work reported by Moonen et al. [31], the final device thickness varies between 60 and 110 µm, which potentially makes a difference in fluidic performance. To prevent this variation, we chose to spin-coat NOA81 on the PDMS mold instead of drop-casting and an application of capillary force to flatten the film. By spin-coating, we assure that NOA81 results in a well-defined thickness on the PDMS mold dependent on the spin-coating speed, yielding consistent final devices. Additionally, given the limitation of our laser source being recently refurbished since our preliminary work described in Moonen et al. [31], we found that making through-holes in an NOA81 foil without a well-defined thicknesses was not reproducible enough within the given ablation parameters of our preliminary work, which mainly focused on the demonstration of the new biological application. Hence, a process window had to be explored more thoroughly. Furthermore, using NOA81 films with well-defined and equal thicknesses, laser ablation within a controlled parameter space must produce micropores with highly reproducible features, including geometry and shape. Only devices made based on defined design specifications will then also yield a well-defined micropore geometry and result in controlled microfluidic and cell capture performance from device to device. Having control over the microfluidic performance of the microsieves is essential, and single cells aimed to be captured inside the pores initially should also be supported throughout the culture time by the microsieve scaffold and kept alive. Single cells might react differently to variant flow speeds and shear stresses. Therefore, it is essential to keep the 3D micropore geometry consistent for repetitive sets of culture experiments by providing devices within circumscribed error margins. To define quality measures in the reproducible performance of the fabrication of NOA81 foils, we measured the resulting foil thickness as a parameter of the spin-speed. Figure 3 shows the data points as well as the fitted spin-curve. These outcomes confirm that fabrication of microsieves with a thickness close to what was reported by Schurink et al. [22] for the original µSEA is now also achievable for NOA81 microsieves. Therefore, spin-coating provides a better condition for comparing the device performance across the fabrication of devices made at different timeslots in the laboratory. Unfortunately, this method of fabrication relies on a piece-by-piece process; hence, variations in the process are critical. One potential challenge in the spin-coating step is that the PDMS mold carries a convex pattern. It is known in the field of microfabrication methods that spin-coating over topography can lead to non-uniform film formation [38,39]. With regard to the features in this work, planarization of the convex patterns was achieved with the selected spin-coat protocol described in the method Section 2.1. Still, minor deviations on film thickness could occur and a cross-sectional study on 3D pore geometry across the rows and columns of the entire microsieve could reveal error margins on film thickness in more detail. In addition, spin-coating results in very flexible foils, which intrinsically lead to difficulties in handling during laser micromachining and cell loading procedure. However, applying assembly to a mechanically stable PMMA ring solves this issue and allows convenient handling and alignment, as can be seen in Figure 2a. A cured NOA81 replica after laser treatment step has been imaged, and the scanning electron micrograph (SEM) is shown in Figure 4. The 900 pyramidal microcavities from the original μSEA have been faithfully replicated into the NOA81 foil. The sharp edges of the pyramidal pores are an indication of a successful replication with high precision. Moreover, the traces of electrodes in the original µSEA with a height of 220 nm were also transferred into the NOA81 foil, which is highlighted in Figure 4 with yellow arrows. This confirms that the added steps to the fabrication process, including plasma treatment and spin-coating, do not negatively affect the replication of even very fine details.

### 3.2. Laser Ablation of NOA81

**Figure 5:**
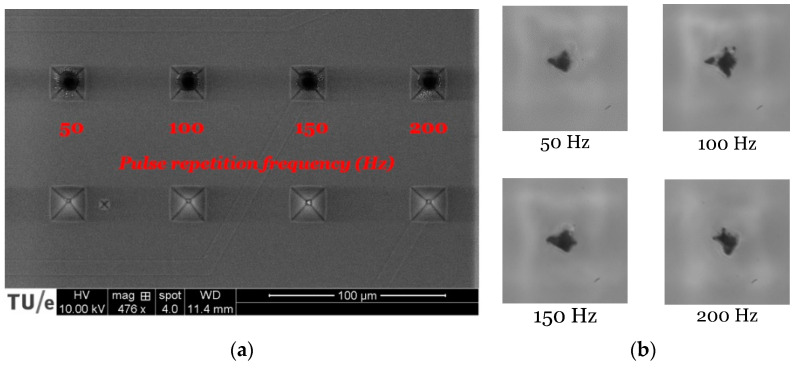
(**a**) SEM top view and (**b**) optical microscopy (×100) bottom view of four different micropores ablated with the same laser parameters except for different pulse repetition rates: 50, 100, 150, and 200 Hz, respectively.

The next step in the fabrication procedure is laser ablation at the tip of the pyramid-shaped microcavities. Achieving a specific 3D micropore geometry depends on the pulse repetition frequency, the laser focus location, and the number of pulses. Pulse repetition frequency refers to the number of pulses sent per second. The influence of the changing pulse rate has not yet been observed in depth, but, in theory, it should affect the surface morphology. Figure 5a shows the quality of the pores for an NOA81 foil spin-coated at 800 rpm, subsequently processed with excimer laser ablation at the center of the microcavities, but at variant pulse repetition rates. The frequency is 50, 100, 150, and 200 Hz. There is not much difference in the quality of the pore geometry and the surface properties in this range of frequencies when inspected by standard top-down SEM or optical microscopy. Therefore, a solid conclusion regarding the influence of repetition frequency on the ablation depth, shape, and produced debris in this laser process cannot yet be drawn. Figure 5b presents the pulse repetition test to compare the exit hole size and geometry of the ablated micropores of an NOA81 foil spin-coated at 800 rpm followed by ablation with 800 pulses at the four different frequencies. Although the exit hole (also called the bottom hole or the aperture of the 3D micropore) results in an irregular shape, there is no other obvious difference. However, the higher the frequency is, the quicker the production will be. This is more critical for the ablation of an entire array of 10 × 10, which will be set as a standard for our future cell capturing experiments or when even more holes should be opened, as this parameter will influence production throughput. Since frequencies above 150 Hz are not recommended for this laser tool, we used 150 Hz for our detailed experiments as an upper limit.

**Figure 6:**
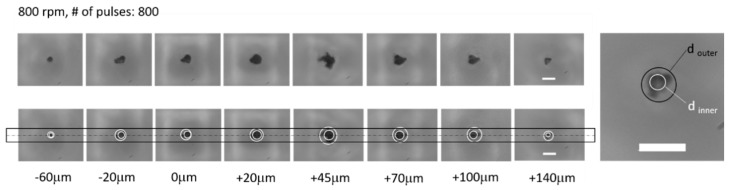
Micropores were ablated with the same laser parameters, except for the vertical distance of the beam focus. This height difference ranged from −60 to +140 µm with 0 as a reference plane. Within steps of 5 μm around a +45 μm off-set, the aperture size maintained an inner diameter between 3 and 4 micrometers (not all results are shown), so a +45 μm off-set from the reference plane was chosen for subsequent experiments.

A parameter of the laser equipment that influences the geometry of the 3D micropore significantly is the laser beam focus. If the laser is well-focused onto the ablation region inside the pyramidal shape, the maximum energy will be given to the surface; hence, ablation will be performed effectively at a minimal number of pulses. On the other hand, if it is focused poorly, the energy will be spread in a larger area, consequently reducing the energy delivered to the part. Since the laser light is beyond the visible wavelength, the LightDeck camera system facilitates the focusing of the laser onto the ablation region. To explore the effect of an off-set in adjusting the focal plane, first the mask patterned was imaged in focus onto the surface of the NOA81 foil using the off-axis light source, and the position was set to Z = 0 as the reference plane. Thereafter, the laser was moved in the Z-direction for a range of distances, and the ablation results were compared to each other. This test was done from Z = −60 µm (meaning the distance of the lens relative to the foil surface was moved down compared to the reference plane) to Z = +140 µm. Figure 6 provides an overview of the results achieved by changing the height of the focal plane in reference to the foil surface. The resulting apertures for each of the settings were observed and imaged by an optical microscope with a ×100 objective. Moreover, Figure 6 presents the aperture diameter size dependent on different positions of the laser, relative to the reference plane and based on a quality definition of the irregular shape. One expects the best result with an outer and inner diameter ratio close to one and an inner diameter between 3 and 4 μm (the design of the through-hole diameter in our application). The results indicate that, for the distance Z between +40 and +50 µm (not all result shown), the inner aperture diameter was in its maximum size. Therefore, +45 μm was chosen for all subsequent laser experiments performing an analysis of the number of pulses against aperture shape.

**Figure 7:**
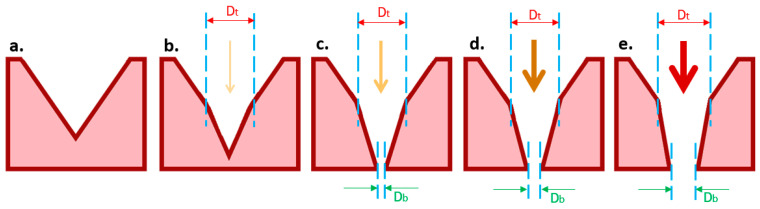
Schematic cross-sectional view of pyramidal-shaped micropores consisting of a combined pyramidal and conical shape to form a 3D micropore. D_t_ represents the diameter of the through-hole top opening made by the laser ablation step in the process, where D_b_ represents the bottom diameter; (**a**) an intact microcavity prior to ablation; (**b**) a low number of pulses may not make a through-hole at all or (**c**) may make a through-hole with a D_b_ that is too small, which is not sufficient for cell capturing; (**d**) for an optimal number of laser pulses, the achieved D_b_ is around 3 to 4 µm; (**e**) a higher number of pulses would widen the size D_b_, which may ultimately result in a poor capturing rate.

An important parameter in laser micromachining is the number of pulses applied to the ablation region. This value should be high enough to produce through-holes but should not make the micropore aperture too wide because cells are very flexible, and with the cells’ diameter being around 10 µm, there is a risk that the cells slip through. Even in holes with a diameter smaller than that of the cell size, cells might deform and squeeze through such micropores in due course of the culture process [40]. Therefore, the fabrication should guarantee a laser exit hole, i.e., an aperture or bottom opening with a size around 3 to 4 μm. Figure 7 shows the sequence of laser treatment where an intact microcavity (Figure 7a) is under laser treatment. If the number of pulses is too low, it may not make a through-hole at all (Figure 7b) or may make through-holes where the diameter of the bottom openings (D_b_) is too small, yielding an overly high flow resistance, which is not sufficient for cell capturing (Figure 7c). If the number of laser pulses is optimal, the achieved D_b_ is around 3 to 4 µm (Figure 7d). A higher number of pulses would then widen the size of the bottom opening, which may ultimately result in a poor capturing rate (Figure 7e).

**Figure 8:**
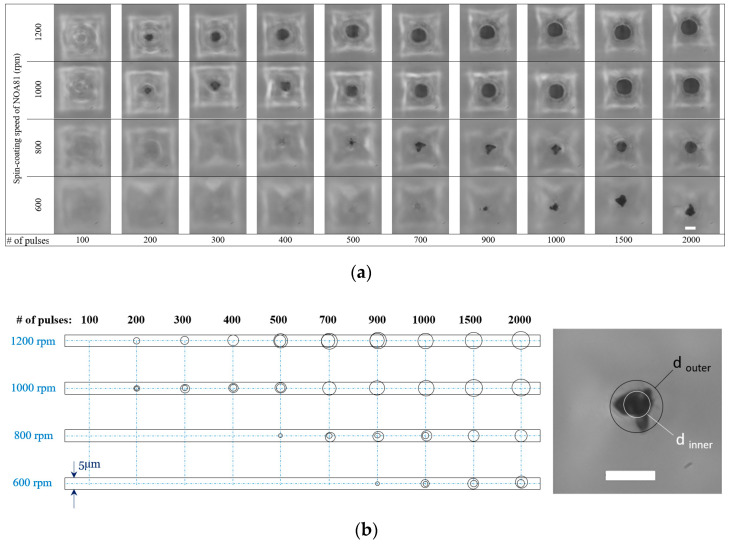
(**a**) The view of the openings on the bottom side of four different NOA81 microsieves. Each has been made with a specific spin-coat speed, which are 600, 800, 1000, and 1200 rpm. On each foil, 14 micropores were realized (selected ones are presented here) with an increasing number of pulses but all with 150 Hz. The scale bar located in the lower right image is 5 µm for all images. (**b**) Spread of the inner and outer diameter of the aperture plotted for each microsieve for an increasing number of laser pulses (the representation of the inner and the outer diameter of a projected circle onto the resulting shape defines the irregularity).

Exploring the influence of the number of laser pulses on the geometry of the ablated region and how the selection of this number affects microsieves with different thicknesses, a set of laser experiments was performed with patterned NOA81 foils that were spin-coated at 600, 800, 1000, and 1200 rpm. On each chip (foil assembled with a PMMA ring), 14 micropores were ablated, each with a different number of pulses but all with the same repetition rate of 150 Hz and with the Z position set to +45 µm. The number of pulses ranged between 100 and 2000. Since, in this experiment, the maximum number of pulses we had applied was 2000 pulses, with the pulse repetition rate of 150 Hz, the required time for any though-hole ablation was 13.3 s. For the chosen range of pulses, we have observed that 100 pulses did not result in any breaching of the foils regardless of their thickness. Figure 8a depicts an overview of the results. As expected, laser ablation with a number of pulses below a certain threshold does not produce a through-hole, which is depicted schematically in Figure 7b. There was an increasing number of pulses above that threshold (for the thinnest NOA81 foils, 200 pulses suffice to start breaching it); however, an increase in the aperture size was observed. In addition, it can be seen from these figures that the aperture area enlarges when thinner foils (i.e., those made with higher spin-coat speeds) are ablated with the same number of pulses as thicker ones. This is a critical point because it shows how important it is to maintain the thickness of the NOA81 foils from device to device within narrow margins, which can be successfully accomplished by replacing the previously applied NOA81 drop-casting step with a spin-coating step in the process. The diameter of each aperture was estimated by projecting an outer and an inner circle onto the resulting shape, as previously depicted in Figure 6. The aperture increased when the number of pulses was increased, as anticipated, but it is important to realize that, if the microsieves are thinner, the previously demonstrated funnel shape, resulting from the laser process, will be lost. The shape of these 3D micropores made by the combination of replica molding and laser ablation will then be more similar to the original geometry realized by silicon micromachining, albeit with a circular aperture rather than a square aperture. This is also shown in the next section via 3D micropore inspection using focused ion beam (FIB)-cut and SEM analysis. These results are similar to our previous laser ablation results in NOA81, as published by Moonen et al. [31]; however, here micropattern fidelity is gained by controlling the NOA81 foil thickness.

### 3.3. Microsieves Characterization by Replica and FIB-Cut Sectioning

**Figure 9:**
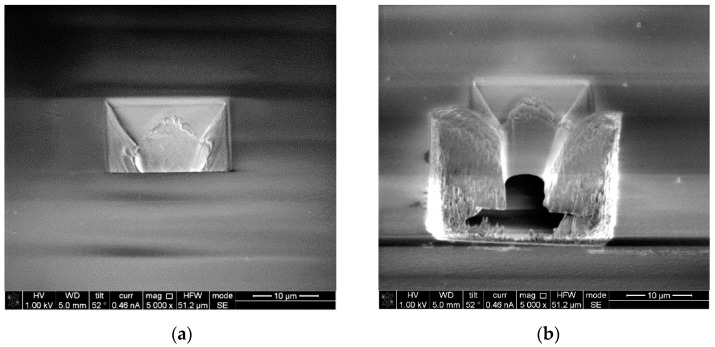
Focused ion beam (FIB) cross-sectional view of a single micropore (**a**) before and (**b**) after FIB-cut sectioning.

**Figure 10:**
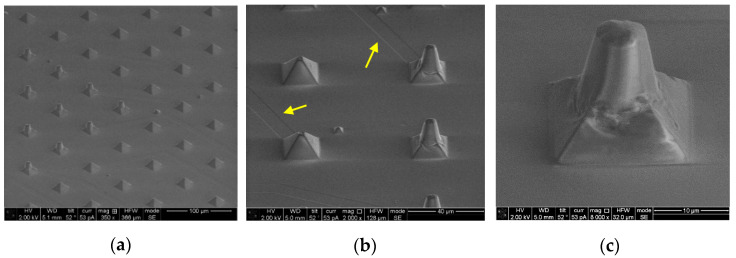
PDMS was cast on a microsieve with an assortment of laser ablated and intact microcavities. The PDMS was then peeled off to demonstrate an inverted copy of the structure; (**a**) presents a wide view of the resulting shapes. (**b**) A closer look at the pyramids where traces of the original 220-nm-thick electrodes are still visible (yellow arrows). (**c**) Close-up of a single PDMS pillar, which delivers a genuine reproduction of the interior surface roughness as well as the surface topography of the 3D micropore.

To examine whether the through-hole was realized with the expected geometry, a cross section view is obtained. This work was done with an SEM/FIB workstation in Nanolab at TU/e with an FEI Nova600i NanoLab (FEI-Thermofisher, Hillsboro, OR, USA). Figure 9 shows a single micropore before and after FIB-cut sectioning. The details of the parameters used in SEM/FIB setup are presented in the Supplementary document. The cross-sectional view of the 3D pore validates not only the control over position but also the geometry of the pore. As expected, the bottom opening was narrower than the top opening, which resembled a funnel-shape through-hole. Although there was no control over reducing the size of the top opening, as this is determined by the laser beam diameter, which was 10 µm, altering laser parameters, most importantly the number of pulses delivered to the ablation region, results in different bottom opening areas. The cross-sectional view analysis made by the FIB-cut tool helped to observe the surface topography on the pore and inside the through-holes, as this is a potential feature whereby neurite outgrowth can be influenced. The inner wall surface of the hole ablated by our KrF excimer laser system appeared to be smooth. Since it is possible that the FIB-cut procedure influences these aspects, an inverted PDMS copy was made to characterize the micropore surface and visualize the topography and geometry of the through-holes. For this purpose, a small amount of PDMS was poured onto a microsieve and peeled off after curing. This inverted PDMS copy of the structure was also inspected by SEM, as demonstrated in Figure 10. Thanks to the high replication capability of PDMS at the nanometer scale (as also confirmed in Figure 4), we expected to transfer the surface relief of the micropore into the copy as well. Figure 10a shows a wide view of the resulting shapes, including pyramids alone and pyramids with pillars. Figure 10b provides a closer look at these shapes, where traces of the original 220-nm-thick electrodes are visible (yellow arrows). Figure 10c provides a close-up of the PDMS copy and shows the inverted shape of a single 3D micropore geometry with the conical through-hole, also visualized by FIB-cut sectioning. The smooth interior surface can be noticed, especially in comparison with the surface roughness on the pyramidal slopes of the 3D micropores, which results from debris produced in the laser ablation step. This might not be the most reliable means of verifying the height of a through-hole because the molded PDMS pillar can be ruptured for any reason during peeling off and thus altering of the height can occur. Still, this procedure provides for a genuine understanding of the interior surface roughness of through-holes as well as the surface topography of the 3D micropores and can be applied for a detailed shape analysis during process optimization. In fact, sectioned views and inverted PDMS copy analysis together provide the means for further analysis of the geometry and surface properties of 3D micropores in the quality control process of manufacturing a larger number of chips. These factors are crucial during cell culturing and neuronal growth, as it is expected that the cells will respond to mechanical cues during the development stage.

## 4. Conclusions

A double replica molding process was employed to fabricate an array of 900 microcavities. In this matrix, individual cavities can be opened in a laser micromachining step to result in a microsieve. Previously, we demonstrated that such a microsieve can be used for pairing single cells to electrodes; here, we wanted to demonstrate that replica molding and the laser ablation process together can be used to fabricate devices within defined error margins. Image analysis of laser ablation tests studying film thickness, the number of pulses, and focus plan variations revealed promising results within a sufficiently wide process window that will also allow for the fabrication of microsieves of a quality fit for a more extensive biological study. This microsieve scaffold-assisted culture platform can now be made with enhanced micropattern fidelity thanks to the use of spin-coating to define the NOA81 film thickness during soft-lithography. This control of NOA81 foil thickness also improves the control of 3D micropore geometry during the through-hole ablation process. Although the laser’s minimal beam diameter is 10 µm, it is shown that, by selecting optimal laser parameters and confining the NOA81 foil thickness, repetitive apertures with a small enough size for capturing cells can be achieved over a relatively large process window. FIB-cut cross-sectional views and inverted PDMS-copy SEM analysis confirmed the desired geometry. The results also demonstrate a reproducible surface roughness and a minimal debris formation on the surfaces of the 3D micropores’ pyramidal slopes and on the side wall of the conical through-hole.

## Data Availability

Data sharing is not applicable to this article.

## Supplementary Materials

The following are available online at https://www.mdpi.com/2072-666X/12/1/21/s1: the details of the parameters used in SEM/FIB setup.
