# Supplementary material for: Gaining Micropattern Fidelity in an NOA81 Microsieve Laser Ablation Process
Source: Micromachines (Basel). 2020 Dec 27;12(1):21. doi: 10.3390/mi12010021 (PMC7823379; doi:10.3390/mi12010021)
Supplement: Supplementary file 1 [file micromachines-12-00021-s001.pdf]

## Supplementary:

To examine whether the through-hole has been realized with the expected geometry, cross section views are obtained. This work has been done in the SEM/FIB workstation in Nanolab at TU/e with a FEI Nova600i Nanolab. Some details of the parameters used in the SEM/FIB setup are as follows:

The tool uses a Ga ion beam for FIB modifications. For the cross-sectioning cut depicted in Figure 9b, the amount of current is set to 6.5 nA. For taking the SEM images shown in Figure 9, the electron beam was at 1 kV in high vacuum mode with a working distance of 5.0 mm while tilted 52°. Moreover, for taking the SEM images of the inverted PDMS copy in Figure 10, the voltage of 2 kV was selected in high vacuum mode.
